# Effect of over-consolidation and shear rate on the residual strength of soils of silty sand in the Three Gorges Reservoir

**DOI:** 10.1038/s41598-017-05749-4

**Published:** 2017-07-14

**Authors:** Deying Li, Kunlong Yin, Thomas Glade, Chin Leo

**Affiliations:** 1 Faculty of Engineering, China University of Geosciences (Wuhan), Wuhan, 430074 Hubei China; 2 Department of Geography and Regional Research, Wien University, Vienna, 1010 Austria; 3 School of Computing, Engineering & Math, Western Sydney University, Sydney, NSW 1797 Australia

## Abstract

Estimation of the residual strength of the soil on the landslide sliding surface is essential for analyzing reactivated landslides. This study investigated the influence of over-consolidation ratio (OCR) and shear rate on the residual strength of SM-type (silty sand) landslide soils in the Three Gorge Reservoir using ring shear tests under drained conditions. A series of ring shear tests were conducted to measure the drained residual strength under over-consolidation ratios of 1–12 and shear rates of 0.06–30.00 mm/min. Test results showed that residual strengths of SM-type landslide soils were not affected significantly by the over-consolidation process. The effect of shear rate on residual strength did not exhibit a regular pattern at shear rates of 0.06–10.00 mm/min, and behaved negatively at a high shear rate of 30 mm/min. The reduction in residual strength at higher shear rates may be attributable to increases in the water content of the shear zone and the amount of finer particles, due to particle breakage and/or larger grains being pushed from the shear zone.

## Introduction

More than 4200 landslides have been observed in the Three Gorges Reservoir area, with consequent loss of life and negative economic impacts^1^. Reactivated landslides represent a very important landslide type in the area. A number of reactivated landslides such as Qianjiangping landslide and Anlesi landslide occurred in the Jurassic strata comprising a series of inter-bedded layers of mudstone, siltstone and sandstone^2^. A typical landslide geological section shows a sliding surface mostly on the contact between Jurassic bedrock and colluvial deposits^3^. In such cases, the shear strength of the landslide soil on the sliding surface is already reduced to a residual state due to repeated sliding and recession. This makes determining the residual strength very important in the analysis of a reactivated landslide.

The residual strengths of landslide soils in undrained and drained conditions have been investigated widely in recent years. For example, the shear-rate effect on residual strength, fluctuation of shear stress, excess pore-water pressure generation and dissipation, localized liquefaction, and shear-zone development processes have been studied using the ring-shear test^4–8^. In ring-shear tests, reconstituted samples of landslide soils, rather than intact samples, have been used extensively to measure shear strength, because it is very difficult to extract intact samples from natural slip surfaces and to determine the direction of field sliding. Reconstituted samples of cohesive landslide soils are allowed to overconsolidate and then are pre-sheared prior to the drained shearing test in order to reduce the testing time and to develop slickensides quickly in the test^9^. However, the effect of overconsolidation on residual strength of SM-type (silty sand) landslide soils may be complex because silty sand is a highly variable soil. Residual strength of SM-type landslide soils may be affected by the loading and unloading processes.

Several previous studies have considered the effects of loading and unloading normal stress on residual strength. Residual strength increased with increasing overconsolidation ratios (OCRs) for Pepper shale and Cucaracha shale^10^.The residual strength of natural soils was independent of stress history and the initial soil condition was not significant influence on residual strength^11^. A considerable reduction in the shear resistance of weathered granitic sand occurred proportional to the total normal stress and shear speed, with finer grains formed in the shear zone due to grain crushing^6^. The effects of artificial overconsolidationon the drained shear behavior of high-plasticity clay soil and sandy-textured loess were investigated. No significant differences in residual strength were found under different overconsolidation ratios^12^.

In the literature, it has been reported that residual strength might or might not vary with different shear rates. Shear behavior during the residual state sheared at higher speeds might exhibit complex shear features. It has been shown that residual strength was not significantly influenced by variation in slower range shear rates, but that qualitative changes in the pattern of shear behavior occurred at rates > 100 mm/min^13^. A significant loss of strength (up to 60% of that for slow shear rates) at a shearing rate >100 mm/min in ring shear tests was found^14^.The residual strength of granular materials was found to be independent of shearing rate^15^. Some researchers have found that residual strength of natural soils is positively dependent on shearing rate^16–18^.

Three types of variation of residual strength occur with increasing shear rate: (1) a positive rate effect—an increase in residual strength when sheared at higher speeds; (2) a negative rate effect—a decrease in residual strength with increasing shearing rate; and (3) a neutral rate effect—a constant residual strength irrespective of shearing rate^19^. Several hypotheses have been proposed in relation to the effect of shear rate on residual strength, such as the occurrence of excess pore-water pressure, increase in water content of the shear zone, frictional heating, liquefaction, mechanical fluidization, and changes to the shear mode and microstructure^17–21^. With regard to the case of the negative rate effect on residual strength, the drastic reduction in residual strength at fast shearing rates may be due to increased water content in the shear zone^19^. In specimens derived from mixed silica sand and bentonite, the shear model and the structure of the shear zone varied with shear rate^22^. Shearing might be controlled mainly by the interactions of sand grains at low shear rates, but at higher shear rates shearing was thought to be controlled by the interactions of clay particles. Both an increase in clay and a reduction of the internal friction angle were found in tested samples compared with the original sample. Therefore, changes in shear strength were thought be due to changes in the geotechnical properties of the samples during shearing^18^. However, the behavior of residual strength for SM-type landslide soils at higher shearing rates and the mechanisms by which shearing rate affects residual strength are not fully understood.

A series of ring-shear tests was performed in the present study using artificially overconsolidated samples subjected to different levels of consolidation stress to understand better the degree of variation in residual strength of SM-type landslide soil under various consolidation processes. Another concern addressed in the present study was the effect of shear rate on the residual strength of SM-type landslide soils loaded with the same consolidation stress. Samples of SM-type landslide soil were collected from a reactivated landslide site in the Three Gorges Reservoir area. The grain-size distribution of the soil samples and the quantities of minerals were examined prior to shearing. Then, a series of multistage ring shear tests were performed. Finally, possible mechanisms by which the over-consolidation ratio and the shearing rate influenced the shear stress-strength behavior were explored.

## Samples and testing procedure

### Ring shear apparatus

**Figure 1:**
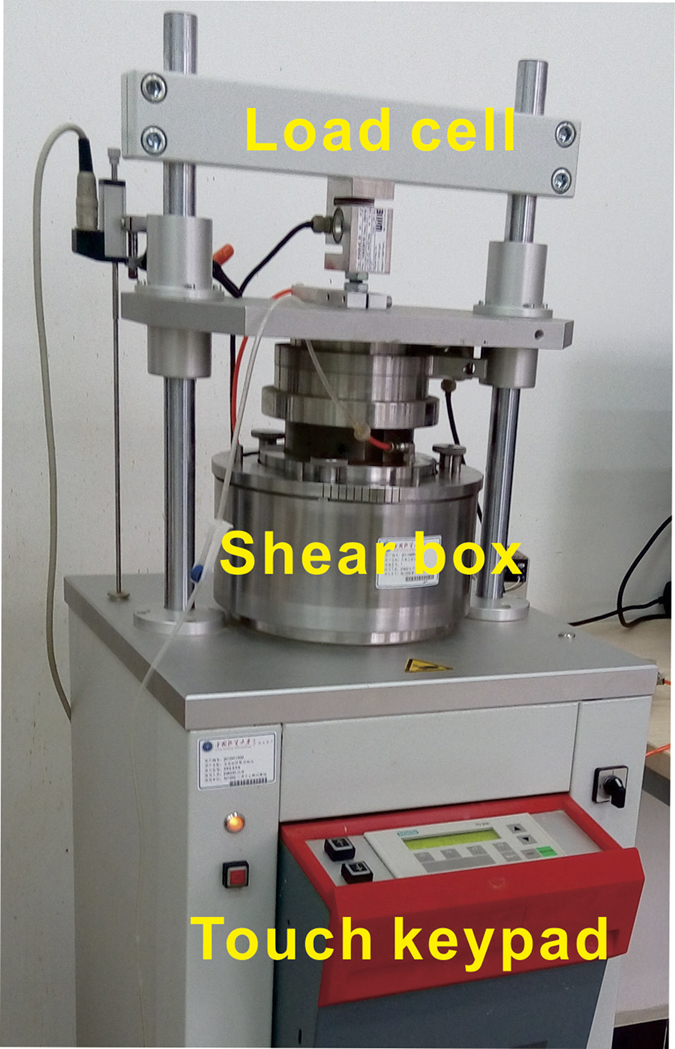
The commercially available soil mechanics ring-shear apparatus used in the study.

The static and cyclic ring-shear apparatus used in this work is shown in Fig. 1. The apparatus is suitable for high-precision ring-shear tests to measure shear strength at peak and residual states using a constant shear plane and unlimited shear displacement. The device could be operated either by a touch-sensitive keypad or by a PC with appropriate software. It had a high-resolution data acquisition system with high-quality transducers for measuring shear stress, normal stress, pore-water pressure, shear strain, and axial strain. The ring-shear box system was constructed of stainless steel, including the upper and lower shear rings, porous ring plates, loading piston, and water receiver(for saturated conditions).

**Figure 2:**
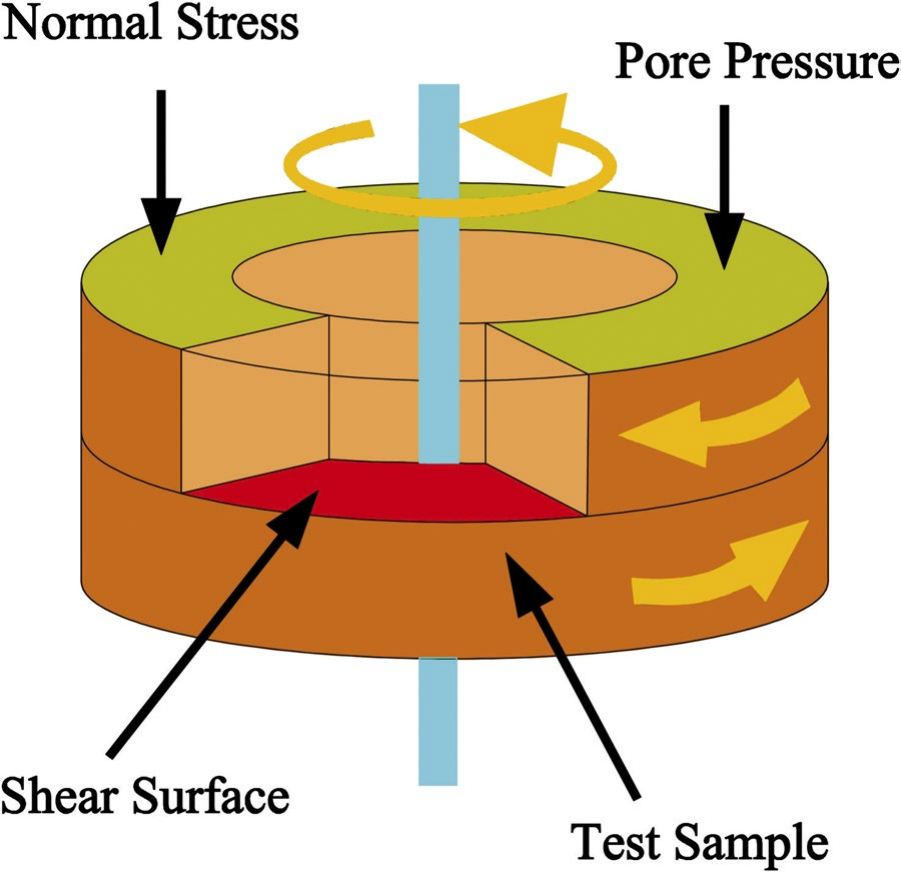
The schematic of the ring shear device.

The interior of the ring shear box had an inner diameter of 100 mm, an outer diameter of 150 mm, and initial thicknesses of 30 mm, giving a shear-surface area of 98.17 cm^2^. The normal stress on the sample was maintained at a constant value during shearing, and the maximum vertical stress was 1000 kPa. The apparatus allowed for application of shear loads up to 10 kN, and vertical displacements up to25 mm. Tests could be run at constant velocity or at constant shear stress. A shear plane was imposed on the mid-height of the specimen by separation between an upper and lower portion of the shear chamber. Excess pore pressure is measured at the top of the sample (Fig. 2).

### Physical properties

**Figure 3:**
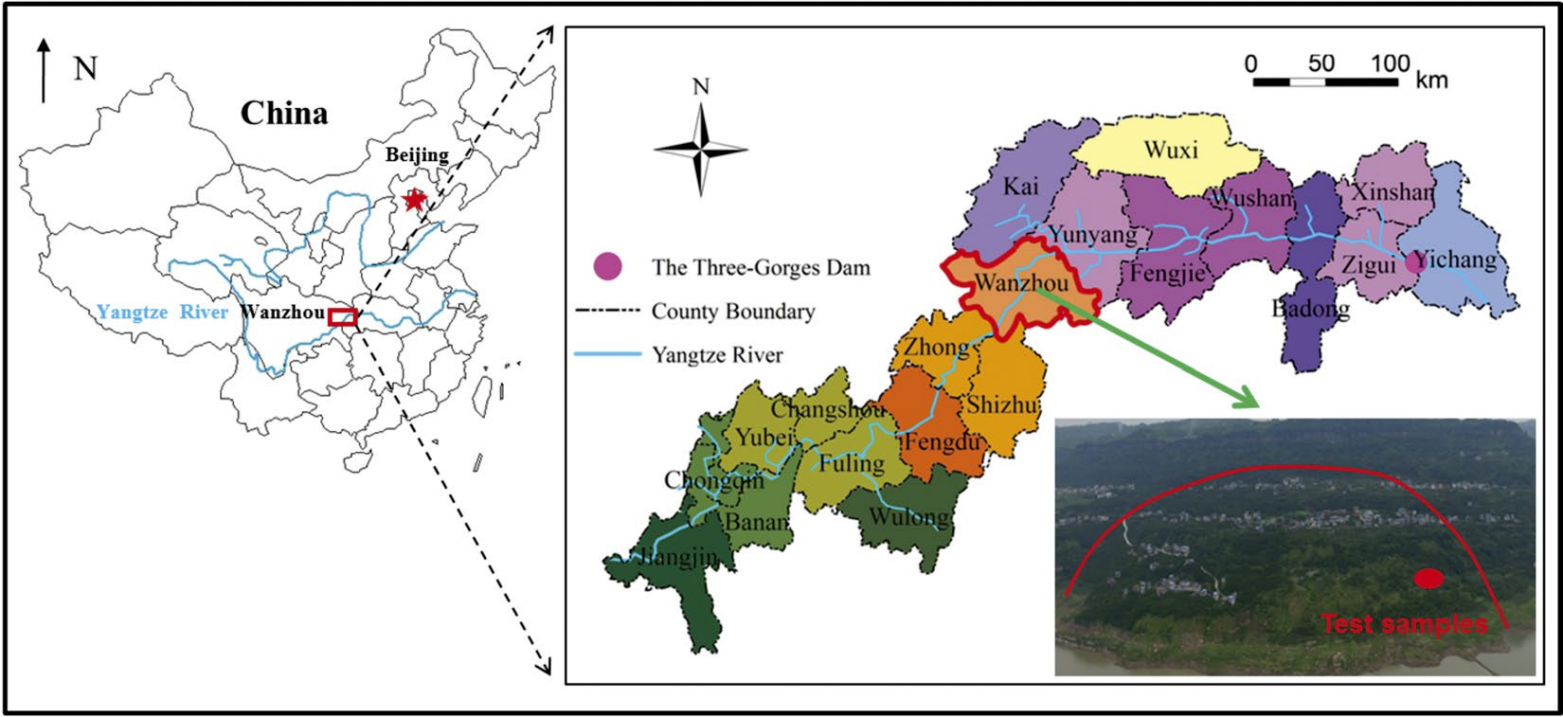
The site of Tangjiao landslide and the location of test samples obtained from the site. The two maps were created by Deying Li in ArcGIS 9.3 software (http://www.esri.com/). The photograph in this figure was taken by Deying Li using a drone.

Test samples were obtained from the failure zones of the reactivated landslide known as Tangjiao landslide at Wanzhou, on the south bank of the Yangtze River in the Three Gorges Reservoir, 270 km west of the Three Gorges Dam. The Tangjiao landslide, is a large, reactivated landslide, with an area of 1.34 km^2^ and an estimated volume of 2672.4 × 10^4^m^3^. The landslide is rectangular in plan with a length of 1020 m and a width of 1300 m (Fig. 3). It extends between 135 and 327 m in elevation, facing the Yangtze River.

**Figure 4:**
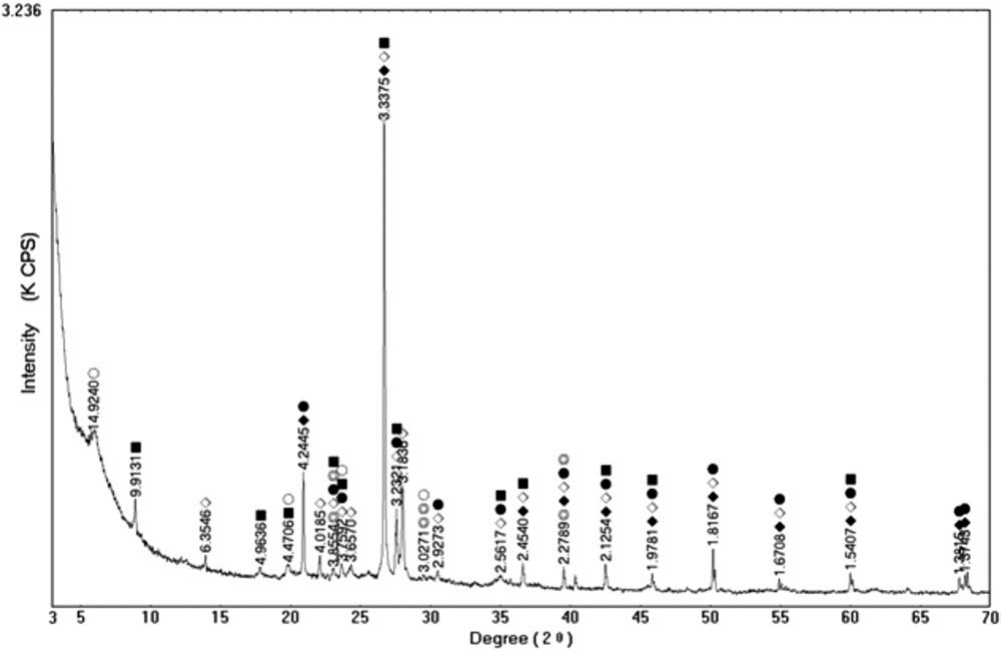
X-ray analysis result.

The samples were air-dried, sieved to exclude particles >2-mm diameter because the maximum particle size permitted in the test is limited to 10% of the initial specimen height^9^. The removed materials comprised 7.6% by weight of the original sample. Grain-size distributions were determined prior to the shearing tests using sieve and hydrometer analyses. The grain-size distribution of a tested sample showed that the proportions of clay (sub-0.002 mm), silt (0.002–0.075 mm), and sand (0.075–2.000 mm) were 7.3%, 26.4%, and 66.3%, respectively^23^. The liquid limit was 31%. The sample was of predominantly sandy texture with a plasticity index of 7%.X-ray powder diffraction was used for quantitative analysis of the proportion of minerals of the sample. Results, (Table 1 and Fig. 4), indicated that quartz and albite were the principal granular minerals, accounting for 85.9% of the sample.

**Table 1:** Proportions of minerals.

Minerals	Quartz	Albite	Illite	Montmorillonite	Orthoclase	Calcite
Proportion	41.42	44.51	10.44	2.07	1.2	0.35

### Testing procedure

Measurements of residual strength were performed using the ring shear apparatus. The ring shear test comprised three basic stages in general: sample preparation stage, consolidation stage, and shearing stage^9^.

### Sample preparation stage

An air-dried representative sample was lightly crushed using a ball mill, and passed through a 2-mm sieve. Reconstituted samples of the sub-2-mm soil fraction were then used to measure the residual strength. De-aired water was added to the processed soil until water content close to the liquid limit was obtained. This was done in order to minimize the amount of air trapped during placement of the soil paste into the annular cavity^9^. The sample was then de-aired in a vacuum chamber with de-aired water for at least 24 h to ensure that it was fully saturated before the sample was placed in the shear box^18^. Samples were surrounded by water in the shear box during consolidation and shearing to prevent them from drying out.

### Consolidation stage

The imposed consolidation stresses used in this study were 600, 500, and 400 kPa. The samples were gradually consolidated in the shear box to the desired consolidation stress (600, 500, or 400 kPa) until completion of primary consolidation was confirmed from the cessation of vertical displacement. Then a shear test to determine the residual strength was conducted at each of the normal stresses of 50, 100, and 200 kPa in an increasing load, multistage shear procedure. Each applied normal stress was held constant during shearing until a steady-state shear stress was obtained, then the normal stress was increased to the next value. For example, shearing was halted when the residual strength was reached at a normal stress of 50 kPa, and then the normal stress was increased to 100 kPa. The sample was then allowed to reconsolidate at 100 kPa before shearing was resumed. After this, the sample was sheared again until a new residual state was reached. This procedure was then repeated for the normal stress of 200 kPa.

### Shearing stage

A series of shearing tests were performed to investigate the shear behavior of silty sand under various over-consolidation ratios and shear rates. Because of the restrictions of the ring-shear apparatus (i.e., pore pressure could not be controlled at fast shear rates), each sample was tested under drained conditions. The residual strength under each application of normal stress was confirmed by the minimum shear stress achieved at steady state. The detailed shear procedure for the different tests is described below.

Effect of over-consolidation on residual strength: To create over-consolidation ratios of 12, 6, and 3, the test sample was first consolidated at 600 kPa. The sample was then unloaded to a normal stress of 50 kPa and allowed to reconsolidate before shearing. The residual strength at 50 kPa was obtained at a constant shear rate of 0.06 mm/min. Then, the procedure was repeated for normal stresses of 100 and 200 kPa. To create different over-consolidation ratios, the same procedure was applied to other samples consolidated to 500 and 400 kPa. Overconsolidation ratios of 10, 5, and 2.5 were created with the 500 kPa consolidation sample. Overconsolidation ratios of 8, 4, and 2 were created with the 400 kPa sample (Table 2). To compare all of the samples, the normally consolidated test series was also divided into three stress combinations of 50/50 kPa, 100/100 kPa, and 200/200 kPa. The initial value of each stress combination represents the effective normal stress in the consolidation phase and the latter value reflects the normal stress in the shearing phase. An appropriate rate of shear was selected based on consolidation data to minimize shear-induced pore-water pressure changes. The choice depended on the estimated shear displacement at failure and total elapsed time to failure: the calculated shear rate was approximately 0.06 mm/min. The sample was sheared at a slow constant shear rate of 0.06 mm/min to ensure the test remained under drained conditions.

**Table 2:** Multistage ring shear procedure.

Test Purpose	Consolidation stress (kPa)	Normal stress (kPa)	OCR	Shear rate (mm/min)
OCR	600(MC1), 500(MC2), 400(MC3), NC(MC4)	50,100,200	12~1	0.06
Shearing rate	600	50,100,200	12,6,3	0.06(MC1), 0.6(MS2), 2(MS3), 6(MS4), 10(MS5), 30(MS6)

MC3 or MS2 indicates test sample number. NC indicates normal consolidation.

OCR is overconsolidation ratio.

**Figure 5:**
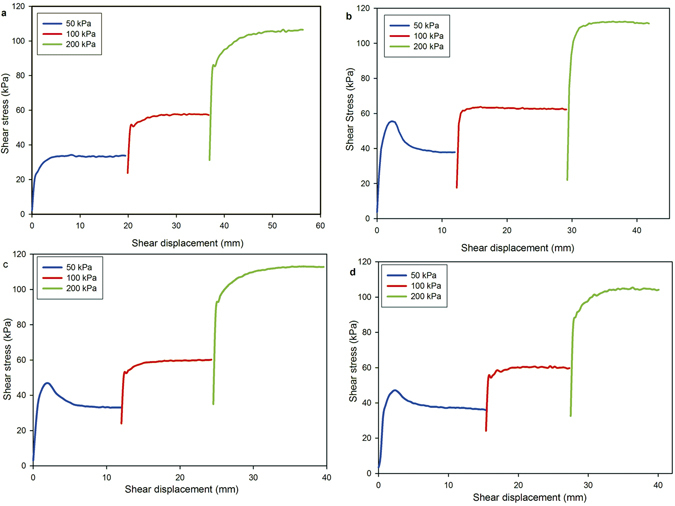
Shear characteristics of the normally consolidated and overconsolidated specimens, (**a**) normally consolidated sample, (**b**) the consolidation stress of 600 kPa, (**c**) the consolidation stress of 500 kPa, (**d**) the consolidation stress of 400 kPa.

Effect of shear rate on residual strength: To investigate the effects of shear rate on the residual strength of over-consolidated samples, a similar multistage ring-shear test procedure was performed at various shearing rates between 0.06 and 30.00 mm/min, but only at 600 kPa consolidation. For these tests, a defined peak in the measured shear resistance was observed at the normal stress of 50 kPa, followed by a gradual decrease in shear resistance to the residual strength. The recorded data showed that a shear displacement of >10 mm was required to reach the residual strength. A typical plot of the residual shear stress versus normal stress for the multistage ring-shear tests is displayed in Fig. 5.

## Test results

### Effect of over-consolidation on shear strength

#### Peak strength and fully softened strength

To distinguish the maximum shear strengths of the specimens under various consolidation conditions in this study, the maximum strength of the normally consolidated specimen was defined as the fully softened strength, and the maximum strength of the over-consolidated specimen was termed the peak strength^24^. Figure 5 shows the shear stress and shear displacement relationships of the normally consolidated and over-consolidated specimens subjected to consolidation stresses of 600, 500, and 400 kPa. The normal stress of 50 kPa, peak strength was obviously larger after consolidation to 600 kPa than that after consolidationsto500 and 400 kPa. However, peak strength did not occur under normal stresses of 100 and 200 kPa because of the pre-existing shear surface from the first shearing under the normal stress of 50 kPa.

As mentioned above, a peak strength occurred only for the normal stress of 50 kPa. Therefore, only the shear characteristics of peak strength for a normal stress of 50 kPa, with different consolidation histories, are discussed in this article. The maximum friction coefficient increased lineally with increasing over-consolidation ratio. The maximum friction coefficient for the normally consolidated specimen increased sharply from 0.675 to 1.099 for an over-consolidation ratio 12 (the sample subjected to the consolidation stress of 600 kPa).The difference in the maximum friction coefficients between the normally consolidated and over-consolidated samples was much more prominent than between the overconsolidated samples with overconsolidation ratios of 8–12, as shown in Table 3.

**Table 3:** Maximum friction coefficient at different over-consolidation ratios (OCRs).

Consolidation stress (kPa)	50	400	500	600
OCR	1	8	10	12
Maximum friction coefficient	0.675	0.926	0.941	1.099

Table 4 shows the horizontal displacements required to reach the maximum friction coefficient during shearing. As shown in Fig. 5, the shear characteristics of the over-consolidated specimens are substantially different from those of the normally consolidated specimen because of the effect of overconsolidation. The maximum strength of the normally consolidated specimen was not obvious. The fully softened shear strength corresponded to the peak shear strength of a normally consolidated specimen. The fully softened friction coefficient was reached after a shear displacement of 8.24 mm. Compared with the shear displacements of the overconsolidated specimens, it was found that the peak friction coefficient with the shortest shear displacement (2.02 mm) occurred for an overconsolidation ratio of 10 (Table 4). The two shear displacements to reach peak strength in overconsolidation ratios 8 and 12 were 2.32 and 2.30 mm respectively. From these results, it is evident that the longest shear displacement required to reach maximum friction coefficient occurred for normal consolidation, and that the amount of shear displacement decreased sharply between an overconsolidation ratio of 1 to an overconsolidation ratio of 8. The shear displacement required to reach the peak strength remained almost constant in the overconsolidated samples.

**Table 4:** Shear displacement required for maximum shear strength.

Consolidation stress (kPa)	50	400	500	600
OCR	1	8	10	12
Shear displacement (mm)	8.24	2.32	2.02	2.3

### Residual strength

**Figure 6:**
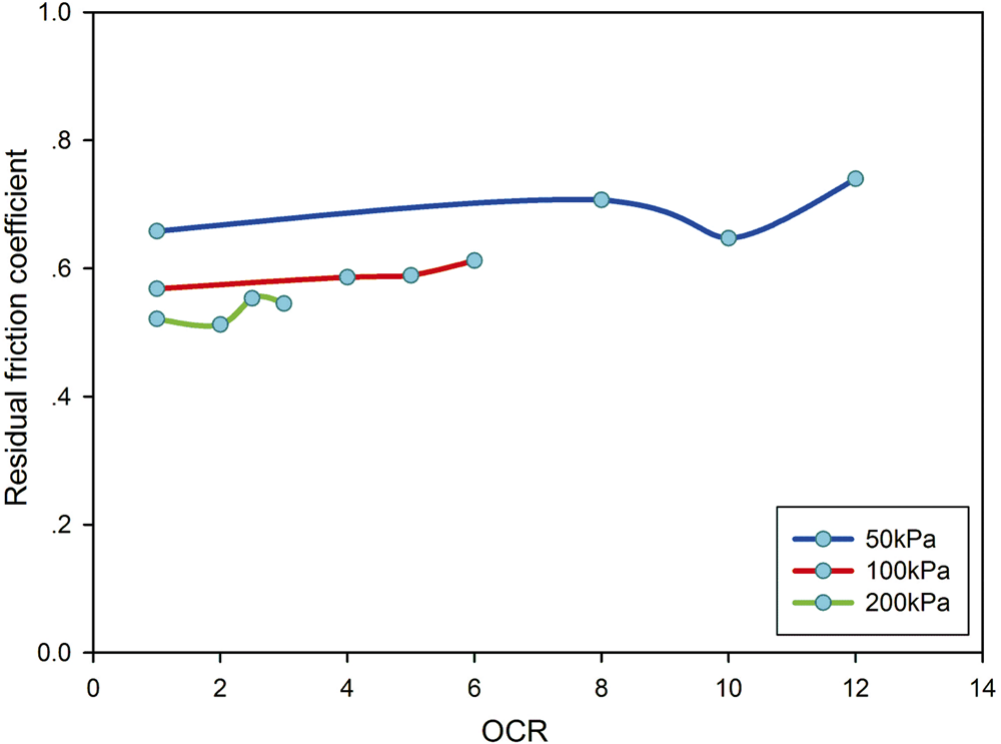
Residual friction coefficient at different overconsolidation ratios (OCRs).

The strength parameters at residual strength were estimated by Coulomb’s law of friction where cohesion was assumed to be zero, following the method described by Skempton^13^. The residual friction coefficient was defined as ${\tau }_{r}/{\sigma }_{n}$. Table 5 compares residual friction coefficients under normal stresses of 50, 100 and 200 kPa. The relationship between residual friction coefficient and overconsolidation ratio is shown in Fig. 6. The residual friction coefficient shows a difference of 0.195 between that for an overconsolidation ratio of 12 (600/50 kPa) and that for 3 (600/200 kPa). The residual friction coefficient for an overconsolidation ratio of 6 was higher by 0.067 than that for an overconsolidation ratio of 3.The differences in residual friction coefficient were greater for different normal stresses. However, it can be seen that for the same normal stress of 100 kPa during shearing, a slight increase in residual strength occurred with increasing overconsolidation ratio, where the maximum recorded friction coefficient difference was 0.044.Under a normal stress of 50 kPa, a slight increase in residual strength also occurred with increasing over-consolidation ratio except at a ratio of 10, where the recorded maximum difference in residual friction coefficient was 0.082. For a normal stress of 200 kPa, there was no obvious tendency in residual strength with increasing overconsolidation ratio. For example, the residual friction coefficient for an overconsolidation ratio of 2.5 was slightly higher (by 0.008) than for an overconsolidation ratio of 3. It was observed that when the specimen was subjected to the same normal stress during shearing, no significant difference in residual friction coefficient was found to be associated with the different consolidation processes. More than half of the test data in the presented data set show only an extremely small change in residual friction coefficient with overconsolidation, suggesting that the effect of overconsolidation on residual strength is not very significant.

**Table 5:** Residual friction coefficients.

Normal stress (kPa)	Consolidation stress (kPa)			
	600	500	400	NC
50	0.740	0.647	0.707	0.658
100	0.612	0.589	0.586	0.568
200	0.545	0.553	0.512	0.521

The variations in maximum shear strength with overconsolidation were more prominent than those in residual strength. The process of loading and unloading appeared to have disturbed the internal particle structure of the high-silt/sand-content samples to cause differences in the peak strength. However, there are two viewpoints regarding the effects of the loading and unloading processes on residual strength: some researchers claim that residual strength is not affected by the initial soil condition^11, 12, 25^, whereas others believe that residual strength is influenced by structural differences^10, 26^. Comparison of residual friction coefficients at the same normal stress during shear shows residual strength was little affected by the overconsolidation process, where the maximum difference in residual friction coefficient between the normally consolidated and the overconsolidated specimens was 0.082. The results suggest that artificial over-consolidation does not cause significant change in residual strength of silty sand. This conclusion that residual strength is not significantly influenced by overconsolidation is in agreement with the findings that residual strength is little affected by initial structure of soil^11, 12, 27^.

### Effect of shear rate on residual strength

**Figure 7:**
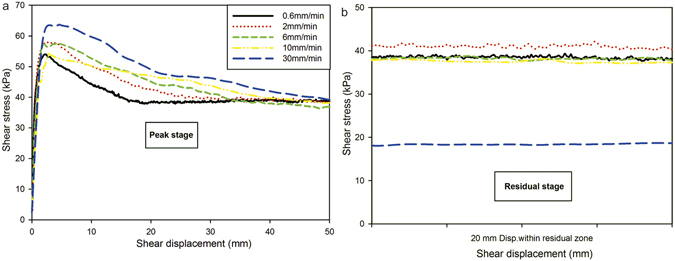
Shear characteristics of the samples at various shear rates at a normal stress of 50 kPa, (**a**) Initial state, and (**b**) 20 mm displacement at the residual state.

The multistage ring-shear test procedure was also performed to investigate the shear behavior of samples under various shear rates between 0.6 and 30 mm/min, for which the normal stresses of 50, 100, and 200 kPa were used during shearing. The shear displacement required from the start of the shear to achieve the residual condition was different under different shearing rates. For the normal stress of 50 kPa during shearing, it was found that the residual condition was achieved easily at a shearing rate of 0.6 mm/min and that the required shear displacement was 52 mm. However, a greater shear displacement was required to reach the residual state as the shear rate increased from 2 to 30 mm/min. At a shear rate of 30 mm/min, the shear displacement required to achieve the residual condition was as much as 2 m. The left side of Fig. 7 shows the shear stress of the test samples during an initial shear displacement of 50 mm at a normal stress of 50 kPa. The right side of Fig. 7 shows a 20 mm continuous segment of the residual friction coefficient curve selected from within the residual zone of the respective sample^12^. The shear rate of the 0.06 mm/min test is not included in Fig. 7 as it is shown in Fig. 5b.

### Determination of residual strength

The value of residual strength under high shear rate was difficult to determine because of the obvious fluctuation of shear resistance in the shearing process from peak strength down to residual strength. A stable residual strength did not occur, even after the specimen was subjected to a shear displacement of 2 m. Moreover, some lower values of shear resistance occurred during the entire shearing process; e.g., at least five lower values of shear resistance occurred after the peak strength at 30 mm/min. For this study, the lowest shear resistance that was maintained at an approximately constant value over a certain period was selected as the residual strength.

### Shear characteristics

**Figure 8:**
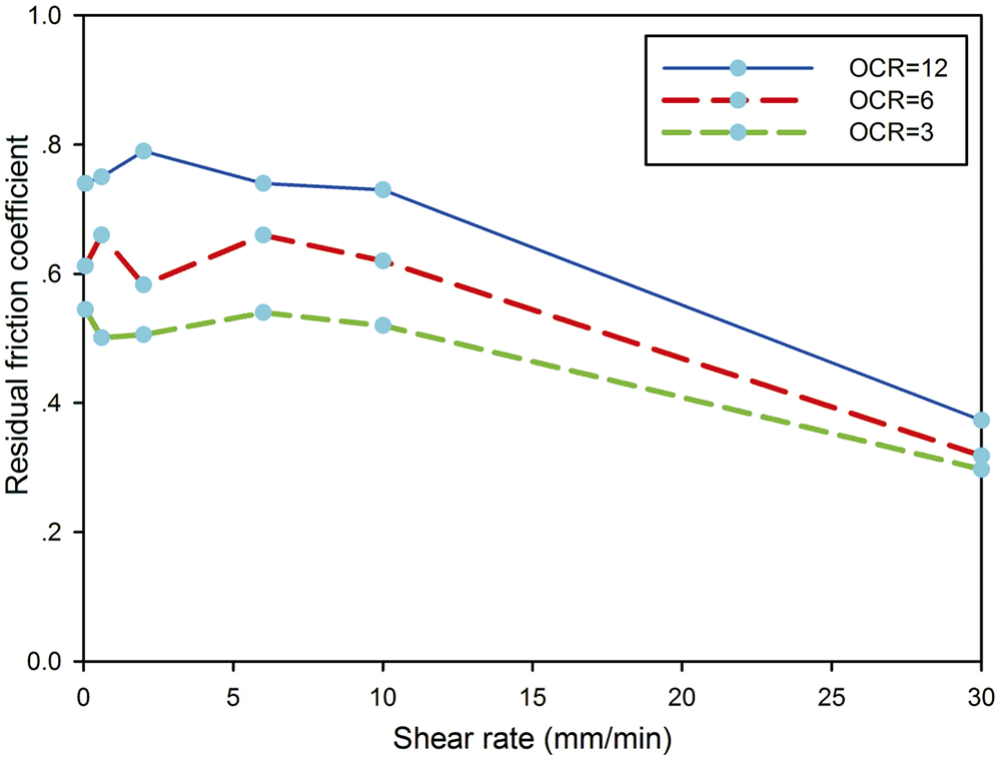
Relationship between residual friction coefficient and shear rate at different overconsolidation ratios (OCRs).

For the study of the effect of shear rate on residual strength, the appropriate strength results for a shear rate of 0.06 mm/min were used in Fig. 8, which shows that the residual friction coefficient varied with shear rate between 0.06 and 30.00 mm/min at different over-consolidation ratios of 12, 6, and 3.For the normal stress of 50 kPa, the residual friction coefficients for overconsolidation ratio 12 were 0.373–0.790. The maximum residual friction coefficient corresponding to 0.790 was at a shear rate of 2.00 mm/min. The differences in residual friction coefficients ranged from 0.00 to 0.06, which was observed as the shear rate increased from 0.06 to 10.00 mm/min. The residual friction coefficient at 0.06 mm/min was equal to that at 6 mm/min. However, as the shear rate was increased to 30 mm/min, the residual strength dropped sharply and the residual friction coefficient reduced to 0.373. As the shear rate was increased from 10 to 30 mm/min, the residual friction coefficient for overconsolidation ratio 12 decreased by 49%. The variation in residual strength at shear rates of <10 mm/min was negligible compared with the difference at higher shear speed of 30 mm/min, and the effect of a high shear rate of 30 mm/min on residual strength was negative.

For overconsolidation ratio 6 under a normal stress of 100 kPa, the residual friction coefficients ranged from 0.318 to 0.660. The differences in the coefficients at shear rates of 0.06–10.00 mm/min were within the range of 0.000–0.077. The maximum residual strength was noted at a shear rate of 0.6 mm/min. The residual friction coefficient at 0.6 mm/min again was equal to that at 6.0 mm/min. The minimum residual friction coefficient was 0.318, which occurred at 30 mm/min. As the shear rate was increased from 10 to 30 mm/min, the residual friction coefficient decreased by 48%. The same phenomena of a sharp decrease in residual friction coefficient and significant negative change in residual strength occurred at higher speeds, although there was no obvious trend in the residual strength with increasing shear rate from 0.06–10.00 mm/min.

For overconsolidation ratio 3 under the normal stress of 200 kPa, the residual friction coefficients were within the range of 0.501–0.545 at shear rates of 0.06–10.00 mm/min. However, as the shear rate was increased to 30 mm/min, the residual friction coefficient reduced to 0.297. As the shear rate was increased from 10 to 30 mm/min, the residual friction coefficient decreased by 43%. It is evident that irrespective of the overconsolidation ratio, the residual strength decreased markedly at the high shear rate of 30 mm/min, and the shearing rate had a negative effect on residual strength. A drastic loss in residual strength was observed at a shearing rate of 30 mm/min. The soil that exhibited the negative effect had a low clay fraction (7.3%), which is similar to the finding of Tika *et al*. for soils with clay fractions ranging from 3% to 55%^19^.

### Fluctuations of shear stress

**Figure 9:**
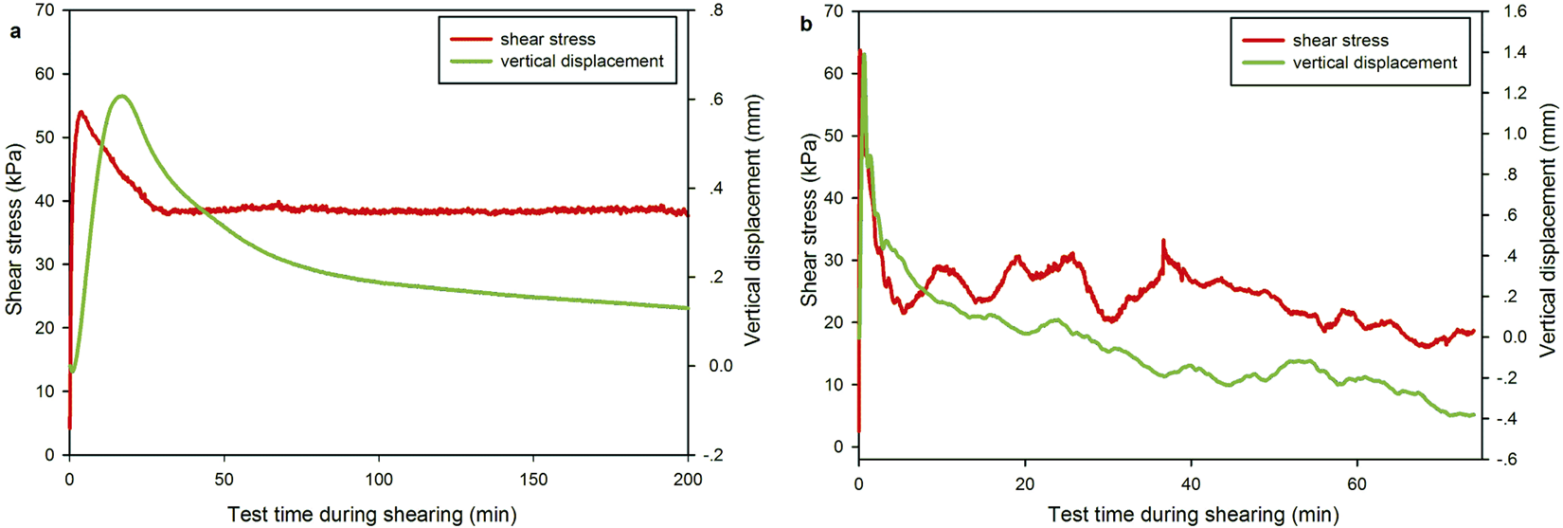
Fluctuations of shear stress and vertical displacement under a normal stress of 50 kPa, at (**a**) a shear rate of 0.6 mm/min and (**b**) 30 mm/min.

Another phenomenon noted in the shear tests were fluctuations in measured shear stress during shearing^28, 29^. The amplitude of such fluctuations for landslide soil with sandy texture increased with increasing shear rate. The samples under a normal stress of 50 kPa during shearing were taken as examples to investigate the characteristics of the shear-stress fluctuations. The sample with the shearing rate of 30 mm/min exhibited high shear resistance of 63.7 kPa and showed strong variability after it had dropped to residual strength (Fig. 9). However, at the shearing rate of 0.6 mm/min, shear-stress fluctuations were negligible and a stable residual state was easily reached. During initial shearing displacement, the vertical deformation dilated because of the overconsolidation. Following the resultant displacement, the sample showed contractive behavior at a shear rate of 0.6 mm/min. Although the general tendency of the sample was contractive under the faster shear rate of 30 mm/min, changes of dilation and contraction were always apparent in the shearing process. Thus, the reason for the fluctuation of shear stress was attributed to the variation in soil volume, where the dilation and contraction of the samples showed strong variability.

## Discussion

### Effect of pore-water pressure on residual strength

**Figure 10:**
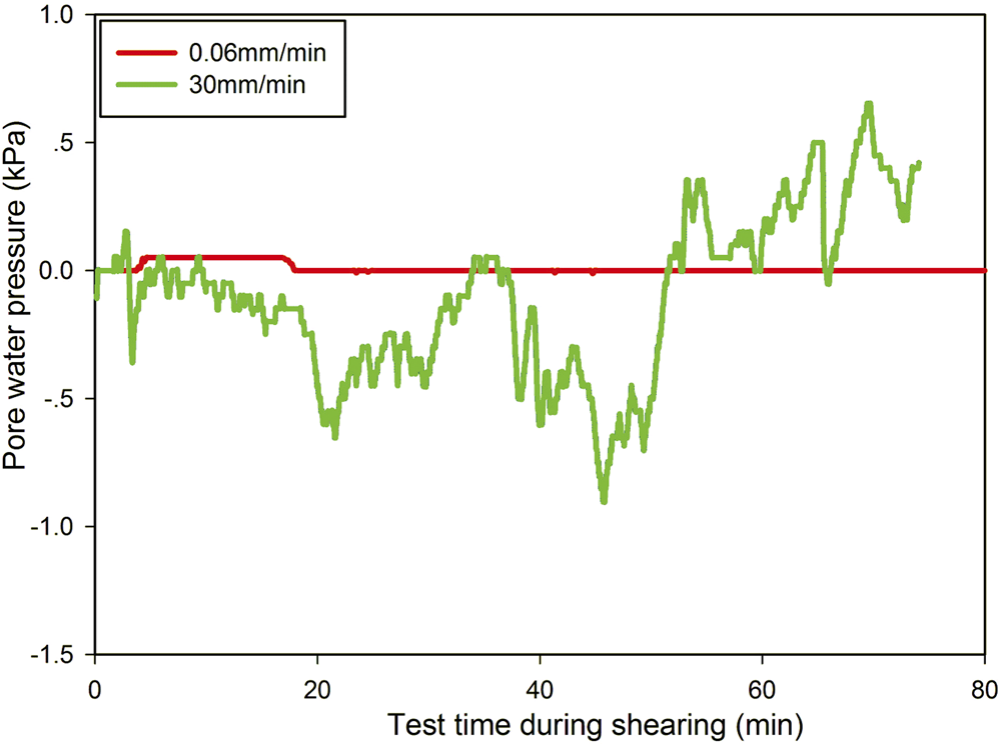
Fluctuation of excess pore-water pressure during shearing under a normal stress of 50 kPa.

In the present study, although the ring-shear tests were carried out under drained conditions, it is possible that some excess pore-water pressure was generated during the fast shearing. The monitored excess pore-water pressures under the normal stress of 50 kPa were taken as examples to investigate pore-water pressures for various shear rates. At a shearing rate of 0.06 mm/min, the excess pore-water pressure remained within a narrow range of 0.05 kPa and could be ignored. The maximum excess pore-water pressure was only 0.65 kPa, which occurred at the fast shear rate of 30 mm/min. All the recorded excess pore-water pressures developed when samples were sheared at various shear rates were less than 1 kPa (Fig. 10). Therefore, the effect of pore-water pressure on residual strength could be supposed negligible.

### Effect of shear rate mechanism on residual strength

A number of mechanisms could explain the results obtained in this experimental study. Firstly, there is the question of whether excess pore pressure in the shear zone could account for the observed behavior. However, in the ring-shear tests, no significant fluctuations in pore pressure were observed and it was assumed that drained conditions existed at all times during shear. Similar results are found by Wang *et al*. ^18^, in ring-shear tests on dry and saturated landslide soils. Positive vertical displacement represents soil dilation and negative vertical displacement indicates soil compression in the curves of Fig. 9. The same trend of shear stress and vertical deformation occurred at the rate less than 10 mm/min was shown in Fig. 9a. However, there were significant fluctuation in shear stress and vertical deformation in the shear process from peak strength to the residual state at a shear rate of 30 mm/min, as shown in Fig. 9b. The recorded vertical displacement showed that fast shearing of 30 mm/min involved larger local dilation, even when the entire tendency was contractive, and that the lower shear strength corresponded to local dilation. This local dilation and loose structure may result in an increase in the void ratio and water content of the shear zone, which could increase the flow capacity of the soil^19^. This may be a factor in how rapid shearing affects the residual strength.

At a shear rate of 30 mm/min, there are significant fluctuations in shear stress even after 2 m of shear displacement (Fig. 9b). Compared to the sample sheared at 0.06 mm/min, the sample was subjected to longer periods of shear displacement and grain crushing. In order to investigate particle breakage qualitatively, the shear-zone samples after the shear test were removed for the grain-size analysis. The grain-size distribution was determined using the laser diffraction method because the samples were too small to sieve^30^. The test result shows that the proportions of clay, silt, and sand after the fast shearing at 30 mm/min were 12.5%, 45.6%, and 41.9%, respectively. Compared with the original sample, the shear-zone sample after shear testing was much finer. Therefore, it is more likely that the sample has increased proportions of finer particles and clay content because of particle breakage, but larger grains may also have been pushed from the shear zone^5, 6, 31^.

Depending on the proportion of platy particles, there are three possible modes of shearing: sliding, turbulent, and transitional^19, 32^. Generally, the turbulent shearing mode occurs in sandy soils because of the high proportion of rounded particles. Clay soils with a high proportion of platy, low-friction particles adopt the sliding mode. The structure of the shear zone and the adopted shear mode might vary with shearing rate^32^. Based on this inference, the turbulent shear mode might change into the transitional or sliding mode because of the increase in finer particles and clay content along the failure zone when a soil is subjected to high shear speed. The change of shear mode from turbulent to transitional or sliding may be another reason for the negative effect on residual strength.

### Reactivated landslides

The laboratory test results show that residual strengths of landslide soils of silty sand texture are not significantly different whether the specimen is overconsolidated or normally consolidated. Therefore, using normally consolidated specimen to measure the drained residual strength of landslide soil in ring shear device is acceptable. The post-failure movement characteristics of reactivated landslides are generally affected by residual strength behavior of sliding surface. In landslide soil with negative rate effect, if a critical combination of landslide displacement and velocity is induced during landslide instability or in limit equilibrium, the shear strength drops sharply, and the landslide accelerates to catastrophic failure and large landslide displacement may occur^33^. This is important in analyzing landslide dynamic characteristics and predicting future landslide movement.

## Conclusions

The influences of both overconsolidation and shear rate on residual strength of sity sand soils were investigated using soil samples obtained from Tangjiao landslide in the Three Gorges Reservoir area. The residual strength was obtained from multistage ring-shear tests conducted under drained conditions. These tests were performed for each sample under normal stresses of 50, 100, and 200 kPa during shearing. To investigate the effect of loading and unloading normal stress on residual strength, consolidation stresses of 600, 500, and 400 kPa were chosen to create different overconsolidation ratios and for comparison with normally consolidated samples. It was found that residual strength was relatively unaffected by overconsolidation.

The effect of shear rate on the residual strength for different overconsolidation ratios was also investigated by increasing the shear rate between 0.06 and 30.00 mm/min. The relationship between residual strength and shear rate did not exhibit a regular pattern at shear rates of < 10 mm/min. However, for samples subjected to the same loading and unloading processes, the shear-rate effect on residual strength was negative with increase of shear rate from 10 to 30 mm/min. The complex shear-rate effect on residual strength might not be influenced solely by an increase in water content in the shear zone, but also by changes of structure in the shear zone and transitions in the shear mode.

## References

[1] Yin YP, Wang HD, Gao YL, Li XC (2010). Real-time monitoring and early warning of landslides at relocated Wushan Town, the Tree Gorges Reservoir, China. Landslides.

[2] Miao HB, Wang GH, Yin KL, Li YY (2014). Mechanism of the slow-moving landslides in Jurassic red-strata in the Three Gorges Reservoir, China. Eng. Geol..

[3] Jian WX, Wang ZJ, Yin KL (2009). Mechanism of the Anlesi landslide in the Three Gorges Reservoir, China. Eng. Geol..

[4] Tika TE (1999). Ring shear tests on a carbonate sandy soil. Geotechnical Testing Journal.

[5] Agung MW, Sassa K, Fukuoka H, Wang G (2004). Evolution of shear zone structure in undrained ring-shear tests. Landslides.

[6] Okada Y, Sassa K, Fukuoka H (2004). Excess pore pressure and grain crushing of sands by means of undrained and naturally drained ring-shear tests. Eng. Geol..

[7] Li YR, Aydin A (2010). Behavior of rounded granular material in direct shear: mechanism and quantification of fluctuations. Eng. Geol..

[8] Schulz WH, Wang GH (2014). Residual shear strength variability as a primary control on movement of landslides reactivated by earthquake-induced ground motion: Implications for coastal Oregon. U.S. J. Geophys. Res. Earth. Surf..

[9] ASTM D6467. Standard test method for torsional shear test to determine drained residual shear strength of cohesive soils, *ASTM International, West Conshohocken, PA* (2013).

[10] LaGatta, D. P. Residual strength of clays and clay-shale by rotation shear tests. Dissertation, *Harvard University* (1970).

[11] Bishop AW, Green GE, Garga VK, Andresen A, Brown JD (1971). A new ring shear apparatus and its application to themeasurement of residual strength. Geotechnique.

[12] Vithana SB, Nakamura S, Kimura S, Gibo S (2012). Effects of over-consolidation ratios on the shear strength of remoulded slip surface soils in ring shear. Eng. Geol..

[13] Skempton AW (1985). Residual strength of clays in landslides, folded strata and the laboratory. Geotechnique.

[14] Tika TE, Hutchinson JN (1999). Ring shear tests on soil from the Vajont landslide surface. Geotechnique.

[15] Hungr O, Morgenstern NR (1984). High velocity ring shear tests on sand. Geotechnique.

[16] Garga, V. K. Residual shear strength under large strains and the effect of sample size on the consolidation of fissured clay. Dissertation, *University of London* (1970).

[17] Bhat DR, Bhandary NP, Yatabe R (2013). Effect of Shearing Rate on Residual Strength of Kaolin Clay. EJGE..

[18] Wang GH, Suemine A, Schulz WH (2010). Shear-rate-dependent strength control on the dynamics of rainfall-triggered landslides, Tokushima Prefecture, Japan. Earth Surf.Process.Landf..

[19] Tika TE, Vaughan PR, Lemos LJ (1996). Fast shearing of pre-existing shear zones in soil. Geotechnique.

[20] Li YR, Wen BP, Aydin A, Ju NP (2013). Ring shear tests on slip zone soils of three giant landslides in the Three Gorges Project area. Eng. Geol..

[21] Hutchinson, J. N. A sliding-consolidation model for flow slides. *Can.Geotech.J*. **23**, 115 ± 126 (1986).

[22] Saito R, Sassa K, Fukuoka H (2007). Effect of shear rate on the internal friction angle of silica sand and bentonite mixture samples. Journal of Japanese Society.

[23] ASTM D422-63. Standard Test Method for Particle-Size Analysis of Soils, *ASTM International, West Conshohocken, PA* (2007).

[24] Terzaghi, k., Peck, R. B., Mesri, G. Soil mechanics in engineering practice, 3^rd^ Ed. Wiley, New York (1996).

[25] Stark TD, Choi H, McCone S (2005). Drained shear strength parameters for analysis of landslides. J.Geotech.Geoenviron. Eng..

[26] Picarelli, L., Leroueil, S., Guerriero, G., Saihi, F. Large deformation shear strength of two types of structured soils. In: AsaokaA, AdachiT, Oka F (Eds.), *Proc. Int. Symp. On Deformation and Progressive Failure in Geomechanics: IS-Nagoya* **97**, 217–222 (1997).

[27] Kock I, Huhn K (2007). Influence of particle shape on the frictional strength of sediments-a numerical case study. Sedimentary Geology.

[28] Tsai JC, Voth GA, Gollub JP (2003). Internal granular dynamics, shear-induced crystallization, and compaction steps. Phys. Rev. Lett..

[29] Fukuoka H, Sassa K, Wang G, Sasaki R (2006). Observation of shear zone development in ring-shear apparatus with a transparent shear box. Landslides.

[30] Kimura S, Kaneko H, Ito T, Minagawa H (2015). Investigation of fault permeability in sands with different mineral compositions (evaluation of gas hydrate reservoir). Energies.

[31] Zhang M, Yin YP, McSaveney M (2016). Dynamics of the 2008 earthquake-triggered Wenjiagou Creek rock avalanche, Qingping, Sichuan, China. Eng. Geol..

[32] Lupini JF, Skinner AE, Vaughan PR (1981). The drained residual strength of cohesive soils. Geotechnique.

[33] Dai FC, Lee CF, Ngai YY (2002). Landslide risk assessment and management: an overview. Eng. Geol..

